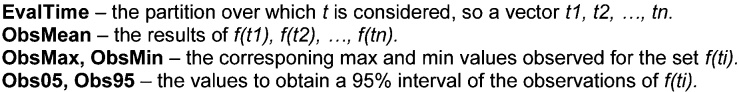# A continuous binning for discrete, sparse and concentrated observations

**DOI:** 10.1016/j.mex.2019.10.020

**Published:** 2019-10-23

**Authors:** Rafael Prieto Curiel, Carmen Cabrera Arnau, Mara Torres Pinedo, Humberto González Ramírez, Steven Richard Bishop

**Affiliations:** a Mathematical Institute, University of Oxford, United Kingdom; b Mathematics Department, University College London, United Kingdom; c Institute for Global Prosperity, University College London, United Kingdom; d École Nationale des Travaux Publics de l'État, ENTPE, Universiteé de Lyon 2, France

**Keywords:** Smooth functions evaluated in concentrated observations, Sparse data, Discrete data, Continuous binning

## Abstract

Discrete observations from data which are obtained from sparse, and yet concentrated events are often observed (e.g. road accidents or murders). Traditional methods to compute summary statistics often include placing the data in discrete bins but for this type of data this approach often results in large numbers of empty bins for which no function or summary statistic can be computed.

Here, a method for dealing with sparse and concentrated observations is constructed, based on a sequence of non-overlapping bins of varying size, which gives a continuous interpolation of data for computing summary statistics of the values for the data, such as the mean.

The method presented here overcomes the problem which sparsity and concentration present when computing functions to represent the data. Implementation of the method presented here is facilitated via open access to the code.

• A new method for computing functions over sparse and concentrated data is constructed.
• The method allows straightforward functions to be computed over partitions of the data, such as the mean, but also more complicated functions, such as coefficients, ratios, correlations, regressions and others.

## Graphical abstract

**Figure:**
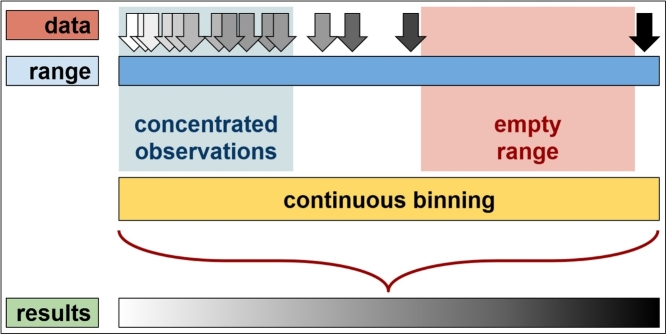

**Specification Table**

**Table:** 

Subject Area:	*Applied Mathematics*
More specific subject area:	*Data techniques and statistics*
Method name:	*Smooth functions evaluated in concentrated observations*
Name and reference of original method:	*NA*
Resource availability:	*Code available at*https://github.com/rafaelprietocuriel/SmoothConcentratedObservations

## Method details

### Introduction

Imagine that there is a cafe, called *Coffeetime*, located in a city centre which is open 24/7. The cafe wants to investigate how much money customers spend at different times in the day. They have records which give the time and the amount paid by each customer. The manager decides to divide the week into one-hour slots and compute the average amount paid by the customers during each hour. However, the manager encounters that choosing one-hour slots (or smaller slots, say 15 min slots), there are some hours for which there are few transactions (during the middle of the night perhaps) and some when there are lots of transactions. For the busy periods, taking the amounts spent over the course of a week, or a month even, to determine average spend, say, is quite straightforward since there are lots of data, but for the quiet times it is not so easy. The manager encounters that there are many hours in which they had little or even no customers and so it is not possible to report the average or the median amount paid by the customers. Infrequent customers might appear just before or after the one-hour slot cut off and so establishing statistics is less meaningful. The manager could consider a coarser partition of the week, for example, to divide it instead of into hours, into groups of two or three hours, in order to avoid empty bins, at a cost of being less precise during the times of the week in which there are many customers. In general, with many types of data, including temporal observations (like the customers from Coffeetime) but also other types of observations (like areas, distances, sizes, volumes, populations and others), dividing the data into groups usually results on empty bins (or slots with no customers).

Beyond visualization of the data [1], a histogram or a density plot is not a sufficient tool for quantifying observations, as simultaneously, the number of customers and the amount of money they spend is being investigated. Thus, there is more than one dimension in the observed data. Also, other cases in which a regular histogram is not sufficient could be relevant, for instance, if data is categorical. If Coffeetime wants to know whether more female or male customers arrive at different times of the day, or their age, their time sparsity and concentration makes traditional methods, such as a histogram, not adequate.

Formally, let the *i*-th observation (customer) arrive at time *ti* in the week (a continuous number between 0, let us say which represents Monday at 0:00, and 168, which represents Sunday night) and let *xi* be the corresponding value of their purchase, which is potentially a vector for each observation (in the case of *Coffeetime*, their receipt could cover the cost of food or just drink or the number of people at that table and more), with *i* between 1 and *n* recorded data (customers). Notice that observations are pairs *(ti, xi)* corresponding to time (*t*) and mark (*x*) for the *i*-th observation.

The manager of Coffeetime wants to know the average amount paid by the customers during different times of the week, meaning that first, a set of observations is filtered (customers which arrived during a specific time interval) and then a function *f* is computed (which, in the case of Coffeetime *f* gives the average amount paid by that group of customers). The function *f* could be more complicated than the average. For instance, *f* could be the ratio of the amount spent by women and by men on the cafe or could be the coefficient of the correlation between the amount paid by the customers and the caloric intake. The function *f* could be as simple as the number of customers but as sophisticated as the manager decides.

#### Bins

The observations are pairs *(ti, xi)* where *ti* represents the time or the variable that wants to be partitioned (in the case of Coffeetime, *ti* is the time of each purchase) and *xi* represents the data corresponding to the *i*-th observation (which is the amount paid, in the case of Coffetime, but could be a list of attributes, including the number of customers of each purchase, their orders, their gender, age and others). Dividing the interval which contains all the *ti* into *k* homogeneous bins, say *b1, b2, …, bk* and then considering separately the set of observations which are contained within each bin, *bj* say (that is, for which the *ti* for *xi* falls within the limits of bin *bj*) allows the function *f* to be computed on that set of observations. In other words, we first, detect which bin the customers are placed in, or at which specific time of the week they spent their money, and then we compute the average spend for every customer over that time period.

Dividing the data into bins is a frequently-used technique, as it conversely allows a discretization of continuous data. This approach has a single parameter, *k,* the number of bins and so it is possible to obtain a more refined or coarser description of the data depending upon the number of bins. In the case of Coffetime, the slots could cover every fifteen minutes or alternatively every two hours with bins typically being of equal size or length.

#### The empty bin dilemma

If non-zero data values were recorded at evenly spaced moments in time, it would be possible to apply existing methods for smoothing of time series, such as moving average, exponential smoothing or local regression models [2]. However, here, one of the challenges is how to deal with the fact that data is sparse and inhomogeneous in time, therefore, there are almost always empty bins. If bin *bj* has no observations, then it is impossible to compute *f* over that set.

**Fig. 1:**
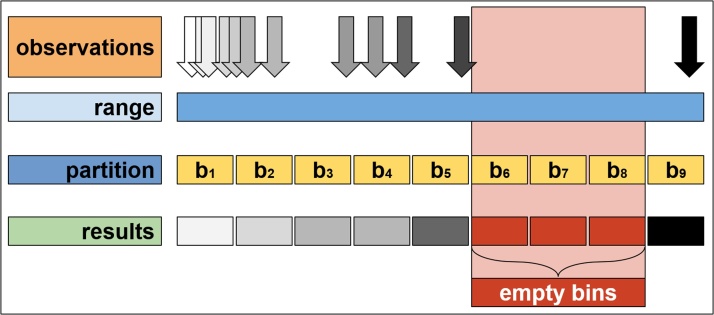
Observations are depicted as arrows, where their position represents the (sparse and concentrated) values of *ti* and the colour represent the values of some variable *xi*, for instance the total value. The function *f* is the “average colour” of the *xi* and so, for empty bins, it is impossible to simply assign a value of the *f(bk)*.

**Fig. 2:**
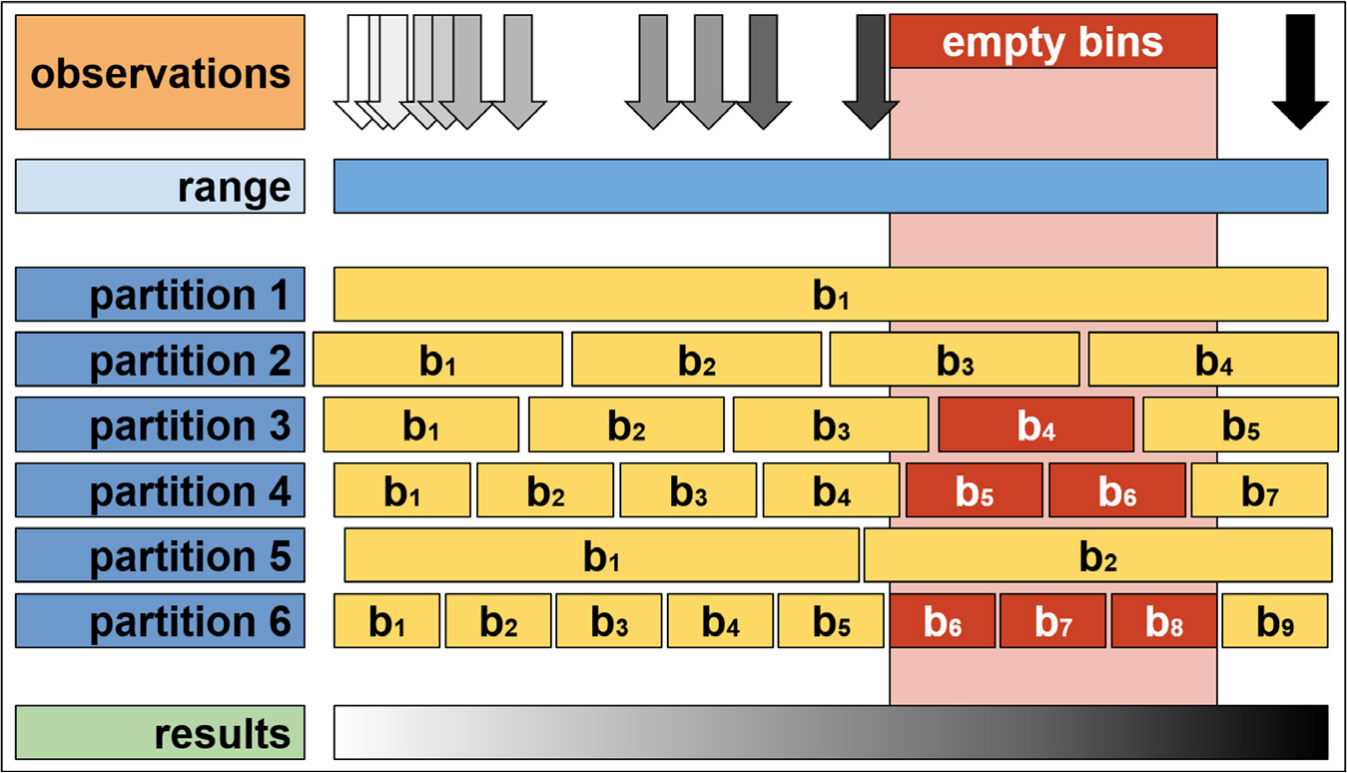
Sparse and concentrated observations are depicted as the arrows, where their position represents the values of *ti* and the colour represent the values of some variable *xi*. Although for different partitions, empty bins are obtained, they are ignored for the computation of the values of *f(tk).* The random initial point of the partitions and the varying width gives a smooth description of the function *f*.

A common technique to deal with empty bins is simply to avoid them by considering a coarser partition of the intervals. Thus, instead of one-hour long bins, two or three-hour bins would be considered, so that there is at least one customer on each bin to compute the function *f*. However, although all of the empty cases are avoided, this technique is clearly not ideal, since very wide bins are too coarse for busy periods. Another option is to map empty bins into missing values and obtain a discontinuous function *f* (Fig. 1, Fig. 2).

This type of challenge is often encountered when considering continuous data which is highly sparse and concentrated. For instance, to detect the Zipf law for Brazilian cities [3], to compare intra-city mobility [4], the temporal patterns of emergency calls [5] or the publication of social media posts [11] and other examples which analyse highly concentrated data, such as power laws [6]. Dividing the data into a uniform partition or a logarithmic binning helps grouping observations with, perhaps, similar attributes and detect its patterns [7].

One interesting example comes from a systematic review of crime concentration at places [8] where the percentage of places (in the horizontal axis) which concentrates a specific amount of crime (vertical axis) is reported for 428 observations or studies around the world. In order to summarize their data, observations were binned into 100 intervals (from 0 to 1%, from 1 to 2% and so on) and the median concentration is computed for each bin. Yet, one of the challenges they encounter is that more than half of their bins are empty, since most studies about concentration focus on very small values of percentage of places, and so most of their data points are concentrated on the left-hand side of the horizontal axis, with very sparse observations on the right-hand side.

A similar issue is actually encountered with the analysis of metropolitan areas in the US [9] *in which their migration patterns were analysed*. Placing, for instance, the population of cities into 400 homogeneous bins (each with a range of 50,000 inhabitants, so *b1 = [50,000; 100,000); b2 = [100,000; 150,000)* and so on) gives 339 empty bins (nearly 85% of the bins are empty). Wider bins have a similar issue. With *k = 80* bins, 68% of them are empty, with *k = 40* bins, 58% of them are empty and even with *k = 15* bins, 40% of them are still empty. Most of the metropolitan areas in the US have a small population, but also there is a large discrepancy between the 9.4 million inhabitants of Chicago, the third largest metropolitan area, and the 18.8 and 19.6 million inhabitants of LA and New York City, the second and first largest metropolitan areas. Thus, empty bins are to be always expected. A logarithmic approach is an alternative for this type of data, where the logarithm of the population is considered (and the size of the bins is now no longer homogeneous). However, with *k = 80* logarithmic bins, still, 24% of the bins are empty.

Data obtained from many social events, such as the size of cities, the number of citations or views of YouTube videos, the frequency of surnames and others, is often best approximated by a distribution which is highly concentrated in some regions, such as a power law or exponential [10], in which case, empty bins are again often obtained, even with a coarse partition.

Here, a method of non-overlapping bins is constructed, for which a refined partition of the range can be considered and no empty bins are obtained, so continuous functions, such as the mean, the maximum or even the coefficients of a regression, are obtained.

### Method

The method consists of an initial “guess” of the function *f* evaluated over the whole range of the data and then, a sequence of partitions or binnings of the range of *t* over which the function is evaluated. Each partition has a varying starting point and width and the function *f* is evaluated, if possible, on each bin, including missing values if the function *f* cannot be evaluated (for example, if given a partition, there are no customers of Coffeetime recorded inside a specific bin, then it is not possible to compute the average value of a ticket). For any given moment, *tj*, the value averaged over all the bins which contained *tj* and which had non-missing values is reported (Fig. 2).

The method for constructing a continuous allocation of observations into bins consists of the following steps:

• Consider an initial allocation that divides the interval under consideration, starting from a selected initial point, into *k* non-overlapping bins denoted by *bi0*, with *i = 1, 2, 3,…, n* each with a width *w0*.
• For each bin, *bi = [ti, ti + 1),* the corresponding observations are identified, such that *tj* falls within *bi*.
• The function *f* is computed for the set of observations, *tj*, obtaining the corresponding *f(bi).*
• If no observations fall within bin *bi* then no value of *f(bi)* is obtained.
• A number *p* of additional non-overlapping allocations into bins, each generated with different random widths and different random starting positions are constructed. For each allocation, the corresponding observations and the corresponding *f(bi)* are computed. Very narrow and very wide bins are considered.
• An allocation consisting of a single bin *b1* is computed, with the corresponding values of *f(b1)* also computed.
• The range of the *ti* is then divided into *m* points, *t1, t2, …, tm*, over which the function *f(tj)* will be evaluated.
• For the point *tk* the corresponding bin *bj* of each one of the *p* allocations and its corresponding *f(bj)* is averaged.
• If the value of *f(bj)* has no value, then it is ignored for computing any metric.
• The new value assigned to *f(tk)* is the average of all the corresponding *f(bj)* where *tk* lies inside each one of the *bj* bins.

It is possible to plot the values of *(tk, f(tk))* as a result of the outlined method.

#### Pseudocode

**Figure:**
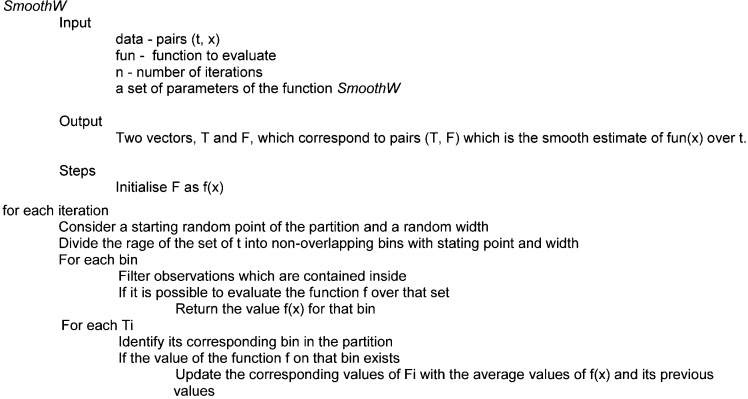

#### Intervals

Notice that for a specific value of *tk,* different values of *f(bj)* are averaged to obtain their mean value. It is also possible to consider departures from that value and construct a 95% interval, which gives departures from the resulting *f(tk)* which would be expected given the observed data.

#### An alternative to the empty bin dilemma

For every value of *tk*, at least one value *f(b1)* is obtained, which comes from the allocation with a single bin *b1*. Thus, even for extreme cases in which data is very sparse or concentrated, a comparison against the overall mean is still obtained. Thus, the method produces a value for every point in the range, and with a sufficiently large value of *p*, that is, with more allocations, local information around the value of *tk* is obtained.

#### Other functions

The way in which the allocations are constructed gives us the ability to consider other functions. For instance, to run a regression for each bin and consider *f(bj)* as the value of one of the coefficients, or the level of adjustment of the regression. The function *f* is potentially more complicated and dependent upon more than one dimension.

### Available code

The code for obtaining is available here: https://github.com/rafaelprietocuriel/SmoothConcentratedObservations

It is possible to compile the code using **R** and based on different functions, *f(bj)*. Although further instructions are included in the code, here we briefly explain its usage.

**Figure:**
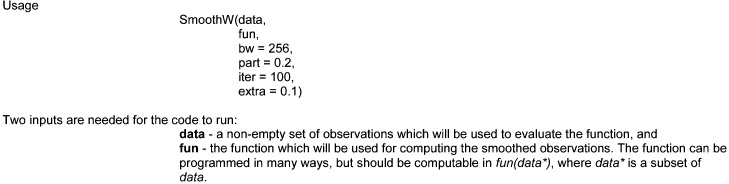

The other parameters of the function are: bw, which is the number of observations which the function will return (with 256 by default); part, a number between 0 and 1, which is the maximum length of the partition for refinement (with a default of 0.2); iter, which is the number of iterations which will run (with a default value of 100 steps); and extra, which is a value which is the number of times that the interval of *t* will be extended on both sides of the spectrum.

The function *f* returns an object, containing the following vectors:

**Figure:**