# Supplementary material for: A continuous binning for discrete, sparse and concentrated observations
Source: MethodsX. 2019 Oct 23;7:100709. doi: 10.1016/j.mex.2019.10.020 (PMC6994295; doi:10.1016/j.mex.2019.10.020)
Supplement: Supplementary file 1 [file mmc1.docx]

**Supplementary material *and/or* Additional information:**

A similar function was used for smoothing the results of a regression coefficient obtained for cities of varying size (Prieto Curiel and others, 2018). In the case of the population in different cities, for instance, tends to have the problem of being sparse and highly concentrated. In the US, for example, metropolitan areas vary from 50,000 inhabitants to nearly 20 million. Yet, there are only two metropolitan areas with more than 10 million inhabitants (NYC and LA) but there are more than 200 metropolitan areas with fewer than 300,000 inhabitants. Thus, regressions computed for sets of cities with varying size have many observations for small cities, but very few observations for large cities. Thus, using the *SmoothW* function gives us the coefficient of regressions for varying sizes of cities.

The function was used in *Temporal and spatial analysis of the media spotlight (Prieto Curiel and others, 2019),* where the time in which tweets were posted is considered the *ti* for *i = 1, 2, …, N,* where *N* is the number of tweets used for the study. Tweets for 28 days were gathered for the study. For each tweet, whether it was related to a specific event (an earthquake in Mexico which occurred on the 19^th^ September, 2017) and whether it was related to a city was analysed. For a set of tweets on a given bin *bi*, the proportion of the number of tweets which were related to the earthquake was computed using the function *SmoothW*, and also, the scaling coefficient of a power law regression where the city-size of the cities mentioned was computed using *SmoothW*. In general, bins which are one-hour long or smaller tend to be empty during the night (meaning, no tweets during periods of one hour) but have enough information during the day (meaning many tweets during the daytime) and so, the problem of *discrete, sparse and concentrated observations* was solved by our method.
